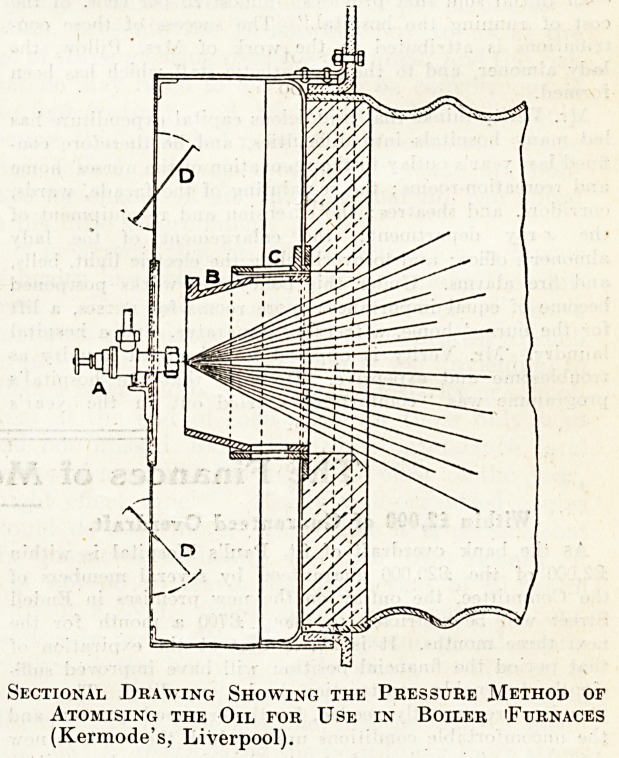# Converting Coal-Burning Boiler Furnaces to Oil

**Published:** 1921-08-20

**Authors:** 


### CONVERTING COAL-BURNING BOILER FURNACES TO OIL.

The use of oil fuel for boiler furnaces has been gradually making its way for a good many years past; partly owing to the steadily rising price of coal, partly to the greater convenience of oil-burning, and partly to saving in labour by converting from coal to oil. This latter consideration would probably not apply in the case of hospital boilers; one man is always necessary for any boiler plant, and he would still be necessary with oil-burning, though he would have considerably less to do. It is in the large boiler plants, for electricity generating stations, very large factories, and, above all, large men-of-war and ocean liners, where the great economies in labour are obtained. At the same time, largely owing to the repeated strikes in the coal trade, and the other troubles that have arisen, boiler-owners are turning more and more to oil. The price of oil threatened to increase very considerably a little while ago, but the enormous deposits which have been discovered in Mexico and elsewhere keep the price down to a figure at which it still compares favourably with coal.

### What Fuel Oil is.

The substance now known as fuel oil has been prepared by the petroleum refiners for some years—since the advent of the Diesel engine. Dr. Diesel intended his engine to burn the pasty mass that was left over from the crude petroleum, the substance as it comes up out of the ground, after everything saleable had been taken out of it; and in the very early engines the pasty stuff was used. Since then the refiners have taken the matter in hand, and produced two kinds of what they call "Fuel Oil," one designed for burning in boiler furnaces, the other for burning in Diesel and semi-Diesel engines. The substance sold under the above name is, the writer believes, really the old pasty mass that used to be thrown away, which has been submitted to a further process of refinement. It is now a liquid, of specific gravity about 0.95, with a flash-point of 150° F., and rather high viscosity, its calorific value being in the neighbourhood of 18,500 B.Th.U., while the calorific value of coal ranges from 10,000 to 15,000 B.Th.U. per lb.; it will be seen, therefore, that a ton of fuel oil has a considerably larger heating value than a ton of coal. In addition a ton of oil fuel occupies 38 cubic feet, while a ton of coal occupies approximately 45 cubic feet; the volume occupied by the coal varies with the quality, the seam from which it is taken, and other matters. Oil fuel, providing it is maintained in the fluid state and not allowed to become too viscous, can be delivered to the boiler furnaces, either by gravity, running down to the furnace from a tank overhead; or, as is usually preferred with large boiler plant, it can be pumped into the furnace from a tank fixed in any convenient position, say, under the boiler-house floor.

### Burning the Oil.

Special arrangements have to be made for burning the oil. As our readers know well, liquids will not burn: it is the vapour produced from the liquid by the aid of heat, and converted into gas by the absorption of further heat, that burns; hence provision has to be made for converting the oil first into vapour and then into gas. The process is rendered very much easier by breaking up the oil into a very fine spray and atomising it. There are several apparatus on the market designed to accomplish this. They may be classed under three systems—air, steam, and pressure; with all of them there is a jet through which the oil is carried well into the boiler furnace. The illustration shows a section of the end of a Lancashire boiler fitted to burn oil fuel by means of a pressure jet. In this case the oil is atomised by the pressure at which it is forced through the jet. As will be seen, the conical spray of atomised oil enters what the inventors call an air cone. A is the jet, and B and C represent the air cone, B being the actual cone whose position can be regulated with reference to C, while D above the jet and D below are louvres, through which air enters, passes up through openings in the cone, and mixes with the jet. With the air system, air under pressure is employed to drive the oil fuel through the jet, to atomise it as it passes into the boiler furnace, and to mix with it, furnishing the necessary oxygen for combustion as soon as the fuel and the air enter the furnace. In the steam system a jet of steam does the same thing with the oil, atomising it and driving it into the furnace as in the air-pressure system. With this system air has to be supplied independently, and the steam for the jet is taken from the boiler.

Sectional Drawing Showing the Pressure Method of Atomising the Oil for Use in Boiler Furnaces (Kermode's, Liverpool).

The simple pressure system is the one that is finding most favour with large boiler plant, but it is probable that the air system would be found more suitable for small plants such as those fixed in hospitals. This latter system can be arranged for coal and oil to be burnt alternately, at convenience; it can also be arranged to burn coal and oil together, to meet special demands for steam. If it is decided to use oil only, the grate bars are removed, and their place taken by fire-brick linings, both above and below. The fire-brick absorbs heat, and assists very considerably in the burning of the oil. Special arrangements should be made for starting up; it is necessary to get everything up to a fairly high temperature before commencing the regular burning of the oil, or else a good deal of soot will be formed. Each apparatus on the market has its own arrangements for doing this.

## Figures and Tables

**Figure f1:**